# Plasma proteomic and metabolomic characterization of COVID-19 survivors 6 months after discharge

**DOI:** 10.1038/s41419-022-04674-3

**Published:** 2022-03-14

**Authors:** Hongwei Li, Xue Li, Qian Wu, Xing Wang, Zhonghua Qin, Yaguo Wang, Yanbin He, Qi Wu, Li Li, Huaiyong Chen

**Affiliations:** 1 Department of Respiratory Medicine, Haihe Hospital, Tianjin University, Tianjin, China; 2 Department of Basic Medicine, Haihe Hospital, Tianjin University, Tianjin, China; 3 Department of Basic Medicine, Haihe Clinical School, Tianjin Medical University, Tianjin, China; 4 Department of Laboratory Medicine, Haihe Hospital, Tianjin University, Tianjin, China; 5 Key Laboratory of RNA Biology, CAS Center for Excellence in Biomacromolecules, Institute of Biophysics, Chinese Academy of Sciences, Beijing, China; 6 Key Research Laboratory for Infectious Disease Prevention for State Administration of Traditional Chinese Medicine, Tianjin Institute of Respiratory Diseases, Tianjin, China; 7 Tianjin Key Laboratory of Lung Regenerative Medicine, Tianjin, China

**Keywords:** Viral infection, Predictive markers

## Abstract

Coronavirus disease 2019 (COVID-19) has gained prominence as a global pandemic. Studies have suggested that systemic alterations persist in a considerable proportion of COVID-19 patients after hospital discharge. We used proteomic and metabolomic approaches to analyze plasma samples obtained from 30 healthy subjects and 54 COVID-19 survivors 6 months after discharge from the hospital, including 30 non-severe and 24 severe patients. Through this analysis, we identified 1019 proteins and 1091 metabolites. The differentially expressed proteins and metabolites were then subjected to Gene Ontology and Kyoto Encyclopedia of Genes and Genomes pathway enrichment analysis. Among the patients evaluated, 41% of COVID-19 survivors reported at least one clinical symptom and 26.5% showed lung imaging abnormalities at 6 months after discharge. Plasma proteomics and metabolomics analysis showed that COVID-19 survivors differed from healthy control subjects in terms of the extracellular matrix, immune response, and hemostasis pathways. COVID-19 survivors also exhibited abnormal lipid metabolism, disordered immune response, and changes in pulmonary fibrosis-related proteins. COVID-19 survivors show persistent proteomic and metabolomic abnormalities 6 months after discharge from the hospital. Hence, the recovery period for COVID-19 survivors may be longer.

## Introduction

Coronavirus disease 2019 (COVID-19), caused by severe acute respiratory syndrome coronavirus 2 (SARS-CoV-2), has emerged as a global pandemic. The disease has been responsible for more than 336 million infections and >5.5 million deaths worldwide by January 19, 2022. Supportive care has been provided for the treatment of patients with COVID-19 [1]. With quick and focused research, a variety of antiviral treatments have been clinically tested and a large-scale COVID-19 vaccination program has been implemented. Thus, the global response to COVID-19 prevention, diagnosis, and treatment has made certain progress [2–6]. However, it is important to investigate the long-term effects of SARS-CoV-2 infection and associated clinical treatments. Patients who survived the SARS pandemic of 2003 were reported to exhibit a poor quality of life 12 years after infection; they were susceptible to lung infections and presented with hyperlipidemia, cardiovascular abnormalities, and other sequelae [7]. Approximately 1 in 10 COVID-19 survivors presented with weakness, palpitation, and dyspnea 3 months after hospital discharge, with half of them exhibiting decreased pulmonary diffusion function [8]. A bidirectional cohort study comprising 1733 COVID-19 survivors indicated that three-quarters of the survivors presented with at least a single typical symptom associated with SARS-CoV-2 infection, such as sleep disorders and psychological problems, in addition to fatigue or muscle weakness, 6 months after hospital discharge [9]. A study of more than 87,000 patients with COVID-19 and nearly 5 million healthy controls showed that the risk of death of COVID-19 survivors in the following 6 months is increased by nearly 60% in addition to the presence of long-term effects affecting almost every system such as the respiratory system, nervous system, mental health, metabolism, cardiovascular system, gastrointestinal system, kidney, blood coagulation regulation, and musculoskeletal system [10]. These studies strongly suggested that systemic alterations continued in a considerable proportion of patients with COVID-19 after hospital discharge. It is thus essential to determine the molecular mechanisms underlying these alterations. Omics technologies, including proteomics and metabolomics, can provide a powerful platform for the study of disease-associated changes in proteins and metabolites in human tissues and fluids, including plasma and urine [11]. Proteomics and metabolomics have important applications in the study of viral infections and communicable diseases [11–13]. The use of proteomics and metabonomic technology can help reveal the pathogenesis of viral infections and provide new targets for the development of treatment strategies and biomarkers. Using these quantitative omics tools, researchers have successfully explored many lesser-known aspects of COVID-19 [14–16]. Tian et al. revealed the occurrence of immunosuppression in patients shortly after SARS-CoV-2 infection, followed by immune overactivation, which contributed to multiple organ damage, especially in patients with severe COVID-19 [17]. The plasma metabolomics of survivors with abnormal pulmonary function derived from COVID-19 is significantly different from that of healthy controls or those with normal pulmonary function after 3 months of discharge [18].

In this study, we used a quantitative proteomic and metabolomic approach to analyze plasma samples obtained from healthy subjects and COVID-19 survivors 6 months after discharge. Proteomic analysis results revealed that the extracellular matrix, immunity, and homeostasis showed abnormalities in COVID-19 survivors compared with healthy subjects. Moreover, metabolomics analysis results indicated the existence of altered lipid metabolism in COVID-19 survivors. These results suggested that long-term recovery might occur in COVID-19 survivors.

## Results

### Demographic and clinical features of COVID-19 survivors 6 months after discharge

**Fig. 1:**
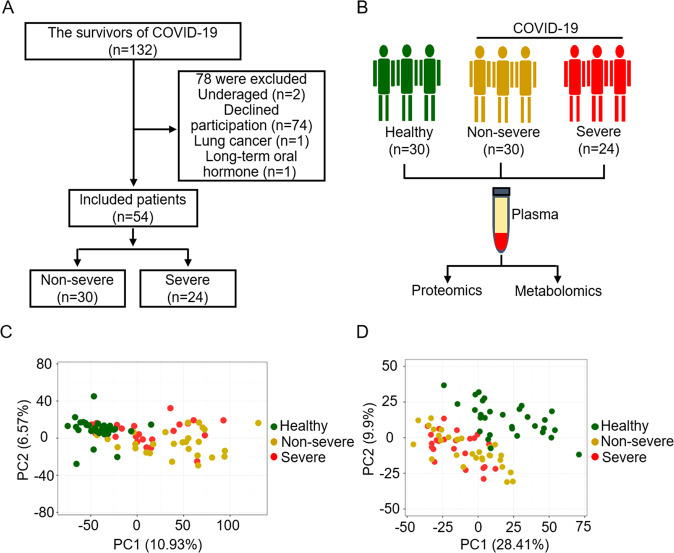
Overview of the study design. **A** Flow chart of inclusion and exclusion for COVID-19 patients enrolled in this study. **B** Schematic summary of the study design and patient cohort. **C** PCA plot of the proteomics data from the plasma samples. Each dot represents one plasma sample, color-coded for the different groups. Green, yellow, and red data points are healthy control subjects and non-severe and severe COVID-19 survivors at 6 months after discharge, respectively. **D** PCA plot of the metabolomics data from the plasma samples. Each dot represents one plasma sample, color-coded for the different groups as described for panel (**C**).

We collected the information on 54 COVID-19 survivors 6 months after discharge, including 30 non-severe and 24 severe patients (Fig. 1A). We observed that the mean age of patients with COVID-19 was 48 ± 15.88 years; 31 patients (57%) were men and 23 patients (43%) were women. The main comorbidity was hypertension (20%), followed by diabetes (13%) and coronary heart disease (9%).

At follow-up examination 6 months after discharge, we found that 22 patients with COVID-19 continued to exhibit at least a single clinical symptom (41%), including 13 severe patients (54%) and 9 non-severe patients (30%). The main symptoms among the patients were fatigue (22%), exertional dyspnea (20%), muscular soreness (17%), and smell and taste dysfunction (9%). Other symptoms included cough (7%), loss of appetite (7%), sore throat (6%), nausea (6%), and abdominal pain and diarrhea (6%). Seven COVID-19 survivors at 6 months after discharge showed an mMRC score of ≥1 (13%), including 1 non-severe (3%) and 6 severe patients (25%). Fourteen COVID-19 survivors at 6 months after discharge showed a Borg score [19] of ≥1 (26%), including 4 non-severe (13%) and 10 severe patients (42%).

In terms of treatment, the proportions of antibiotics and hormones used in severe patients (96%, 42%, respectively), were higher than that in non-severe patients (43%, 7%, respectively).

We also matched 30 healthy subjects with similar demographic characteristics for establishment of the healthy control group. The demographic characteristics and clinical symptoms of the 54 patients and 30 healthy controls have been summarized in Table 1. Thirteen COVID-19 patients (26.5%) still had chest high-resolution computed tomography abnormalities at 6 months after discharge, including 1 non-severe patient (3.6%) and 12 severe patients (57.1%). The difference between the two groups was significant (Table 2).

**Table 1:** Demographics and Clinical Characteristics of COVID-19 Patients 6 months after discharge and Healthy Control.

Characteristics	Healthy Control (*n* = 30)	COVID-19		
		Total (*n* = 54)	Non-sever (*n* = 30)	Severe (*n* = 24)
Sex - no. (%)				
Male	17 (57%)	31 (57%)	17 (57%)	14 (58%)
Female	13 (43%)	23 (43%)	13 (43%)	10 (42%)
Age - year				
Mean ± SD.	46 ± 6.47	48 ± 15.88	43 ± 15.18	55 ± 14.28
Range	36–59	19–89	19–89	28–79
Smoke - no. (%)		5 (9%)	2 (7%)	3 (13%)
Alcohol - no. (%)		5 (9%)	2 (7%)	3 (13%)
Comorbidity - no. (%)		18 (33%)	6 (20%)	12 (50%)
Hypertension		11 (20%)	5 (17%)	6 (25%)
Diabetes		7 (13%)	1 (3%)	6 (25%)
Coronary heart disease		5 (9%)	0 (0%)	5 (21%)
Symptoms - no. (%)		22 (41%)	9 (30%)	13 (54%)
Fatigue		12 (22%)	5 (17%)	7 (29%)
Exertional dyspnea		11 (20%)	4 (13%)	7 (29%)
Muscular soreness		9 (17%)	4 (13%)	5 (21%)
Smell and taste dysfunction		5 (9%)	1 (3%)	4 (17%)
Cough		4 (7%)	2 (7%)	2 (8%)
Loss of appetite		4 (7%)	0 (0%)	4 (17%)
Sore throat		3 (6%)	3 (10%)	0 (0%)
Nausea		3 (6%)	1 (3%)	2 (8%)
Abdominal pain and diarrhea		3 (6%)	1 (3%)	2 (8%)
mMRC score				
0		47 (87%)	29 (97%)	18 (75%)
≥1		7 (13%)	1 (3%)	6 (25%)
Borg score				
0		40 (74%)	26 (87%)	14 (58%)
≥1		14 (26%)	4 (13%)	10 (42%)
Treatment - no. (%)				
Antibiotics		36 (67%)	13 (43%)	23 (96%)
Antiviral drug		53 (98%)	29 (97%)	24 (100%)
Chinese medicine		53 (98%)	30 (100%)	23 (96%)
Hormone		12 (22%)	2 (7%)	10 (42%)

**Table 2:** Comparison of Chest HRCT between Non-severe and Severe Groups 6 months after discharge.

Chest HRCT	Non-severe (*N* = 28) (%)	Severe (*N* = 21) (%)	*p* value
Abnormal	1 (3.6%)	12 (57.1%)	2.63 E-05

### Plasma proteomic and metabolomic profiling of COVID-19 survivors 6 months after discharge

We used the data-independent acquisition (DIA) proteomics strategy and targeted metabolomics approach to analyze undepleted plasma from 84 individuals (Fig. 1B). Overall, 1019 proteins were quantified and 1091 metabolites were identified through a compound library search. The coefficient of variation (CV) values of 92% of proteins were demonstrated to be <30% (Fig. S1A) in QC samples and meanwhile, the CV values of 82 and 95% metabolites were <20 and 30%, respectively (Fig. S1B). The median CVs for the proteomic and metabolomic data were 12 and 10%, respectively (Fig. S1C). These results indicate that the MS data were highly consistent and reproducible. PCA of quantified proteins revealed that there were significant alterations in the COVID-19 survivors’ group (including severe and non-severe group) compared to the healthy controls (Fig. 1C). However, the distinction between severe survivors and non-severe survivors among COVID-19 patients was not significant. Metabolome analysis also showed that the COVID-19 survivor and healthy groups were obviously distinct, and the difference between the non-severe and severe groups was relatively small (Fig. 1D).

**Fig. 2:**
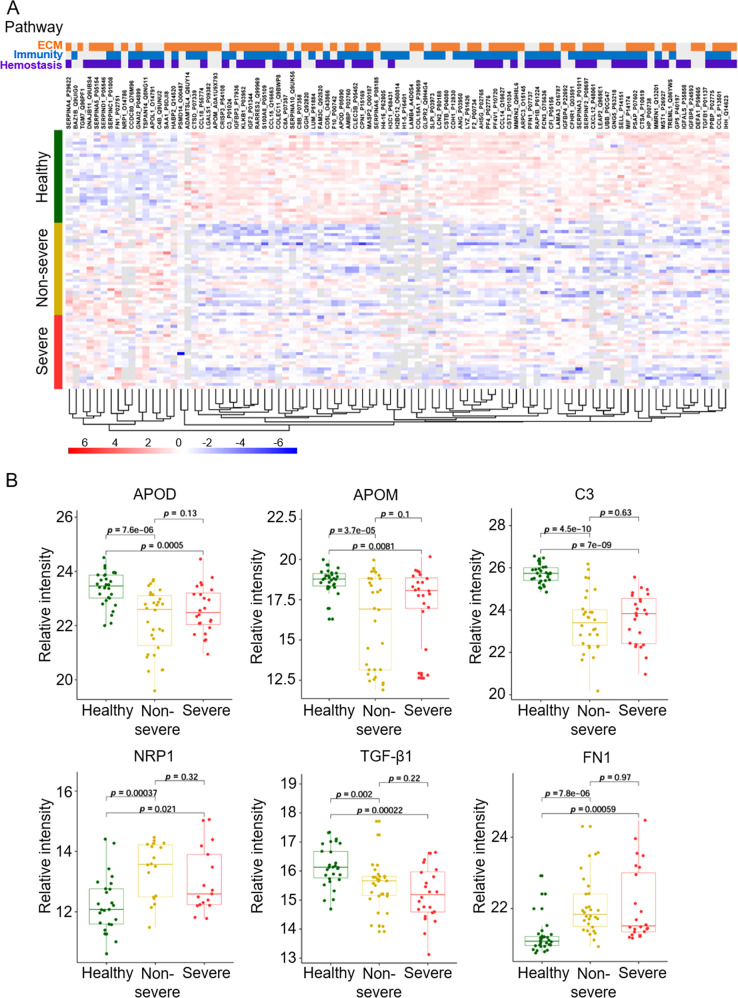
Proteomic profiling of plasma samples obtained from COVID-19 survivors 6 months after discharge and healthy control subjects. **A** Heatmap visualization of significantly differentially expressed proteins (DEPs) whose regulation concentrated on three enriched pathways. The graphs show the relative intensity of DEPs. Proteins included in the heatmap meet the requirement that fold-change >1.5 or <0.67 and *p* value (*t* test) of <0.05. *p* values were then adjusted using the Benjamini-Hochberg correction (false discovery rate, <0.05). The color bar represents the relative intensity of identified proteins from −6 to 6. **B** The boxplots show six proteins, which are significantly different between COVID-19 survivors 6 months after discharge and healthy control subjects. Healthy group, *n* = 30; non-severe group, *n* = 30; severe group, *n* = 24. APOD apolipoprotein D; APOM apolipoprotein M; C3 complement 3; FN1 fibronectin 1; NRP1 Neuropilin-1; TGFβ1 transforming growth factor beta 1.

A total of 272 differentially expressed proteins (DEPs) were identified between the COVID-19 survivors (including severe and non-severe) and healthy control group, of which 35 were up-regulated and 137 were down-regulated (Fig. S2A). The result of PCA analysis indicated that the DEPs generated by the comparisons could effectively distinguish between the COVID-19 survivors and the healthy controls (Fig. S2B). In the COVID-19 survivors, the heterogeneity of patients is large, and it is difficult to distinguish between severe and non-severe patients. To further understand the function of DEPs and their involvement in biological processes, GO biological processes (BP) and Reactome gene sets were applied using Metascape (Fig. S3 and Table S4). The top 20 enrichment function terms showed that the DEPs in COVID-19 survivors compared to in healthy controls were mainly involved in biological processes associated with the extracellular matrix (ECM), immunological response, and hemostasis (Fig. S3A). The ECM pathway includes extracellular structure organization, collagen metabolic process, and regulated exocytosis. Immunological responses include humoral immune response, leukocyte migration, acute-phase response, and initial triggering of complement. Hemostasis includes the formation of a fibrin clot (clotting cascade) and complement and coagulation cascades. The network result of enriched function terms showed that the functions of hemostasis, immunological responses, and ECM are closely related (Fig. S3B), and some key proteins are involved in these processes. Heatmap visualization of the DEPs revealed that three main pathways were enriched (Fig. 2A).

**Fig. 3:**
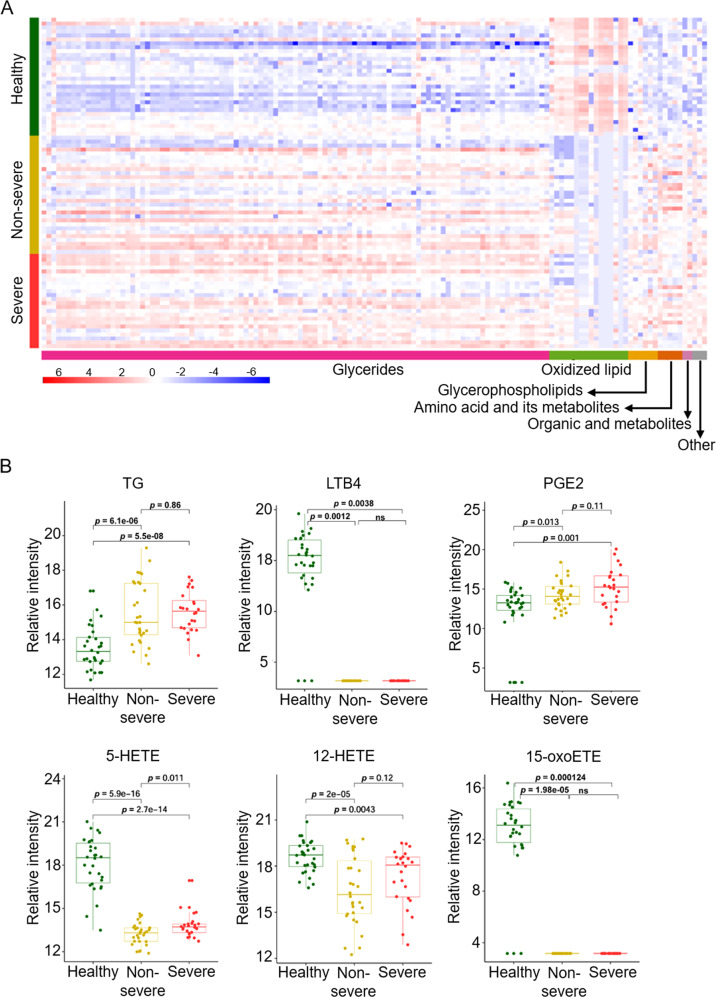
Metabolomics profiling of plasma samples obtained from COVID-19 survivors 6 months after discharge and healthy control subjects. **A** Heatmap visualization of significantly different altered metabolites (DEMs) in COVID-19 survivors at 6 months after discharge and in healthy control subjects. Metabolites included in the heatmap showed a fold-change >2 or <0.5 and *p* value (*t* test) of <0.05. The color bar represents the relative intensity of identified proteins from −6 to 6. **B** Boxplots of six elected metabolites that significantly differed between COVID-19 survivors at 6 months after discharge and healthy control subjects. For the healthy control group, *n* = 30; for the non-severe group, *n* = 30; for the severe group, *n* = 24. 5-HETE 5-hydroxyeicosatetraenoic acid; 12-HETE 12-hydroxyeicosatetraenoic acid; LTB4 leukotriene B4; 15-oxoETE 15-oxoeicosatetraenoic acid; PGE2 prostaglandin E2; TG triglycerides.

In COVID-19 survivors compared with healthy controls, 453 metabolites with VIP values >1 accounted for 41.52% of all metabolites detected. Then, according to the screening criteria for significantly differential metabolites (DEMs) discussed in the Methods section, a total of 135 significant DEMs were detected between COVID-19 survivors and healthy controls, of which 118 were up-regulated and 17 were down-regulated (Fig. S4A). The results of PCA were similar to those of plasma proteomics, and the DEMs generated by the comparisons effectively distinguished between COVID-19 survivors and healthy controls (Fig. S4B). These DEMs included glycerides (76.30%), oxidized lipids (11.85%), glycerophospholipids (GP, 4.44%), and amino acids and their metabolites (3.70%) (Fig. S4C, Table 3). The results of the top 20 metabolites with high VIP values are shown in Fig. S4D. Heatmap visualization of the DEMs showed the altered metabolites (Fig. 3A).

**Table 3:** The Class of significantly different metabolites.

Class	Count	Proportion (*n* = 135)
Glycerides	103	76.3%
Oxidized lipids	16	11.85
Glycerophospholipids	6	4.44%
Amino acid and Its metabolisms	5	3.70%
Organicacid and Its derivatives	2	1.48%
Other	3	2.22%

### Altered lipid metabolism and disordered immune responses

Previous research showed that immunological responses and hemostasis were associated with pathological processes in patients with COVID-19 [20–24]. Our results suggested that abnormalities in those pathways or processes persisted in COVID-19 survivors 6 months after discharge and were thus considered to be responsible for the occurrence of persistent symptoms or increased risk associated with the symptoms reported in those patients after recovery [9, 25]. As an important constituent of humoral immunity, the complement system is known to play a crucial role in COVID-19 infection. The present study highlighted a decrease in the levels of complement C3 in COVID-19 survivors 6 months after discharge (Fig. 2B). Interestingly, a retrospective cohort study showed that reduced levels of complement C3 were associated with poor prognosis in patients with COVID-19 [26]. Neuropilin-1 (NRP1) is a host factor involved in the establishment of SARS-CoV-2 infection and has been reported to promote SARS-CoV-2 cell entry and increased infectivity [27, 28]. Our study found that the levels of NRP1 were higher in COVID-19 survivors compared with those in healthy controls (Fig. 2B). Both leukotriene B4 (LTB4) and prostaglandin E2 (PGE2) are derived from arachidonic acid and have been demonstrated to affect the antiviral immune response [29]. Our analysis revealed the downregulation of LTB4 expression and upregulation of PGE2 expression in COVID-19 survivors (Fig. 3B).

Levels of a variety of polyunsaturated fatty acid metabolites, including 5-hydroxyeicosatetraenoic acid (5-HETE), 12-hydroxyeicosatetraenoic acid (12-HETE), and 15-oxoeicosatetraenoic acid (15-oxoETE), were downregulated in COVID-19 survivors (Fig. 3B). Particularly, 5-HETE, which is the major metabolite produced by 5-lipoxygenase, is involved in the biosynthesis of human leukocytes [30, 31]. Furthermore, 12-HETE has been shown to exert anti-inflammatory activity and to block the TNF-α induced secretion of IL-6 by macrophages [32, 33]. Changes in the levels of 12-HETE have also been observed in animal models of influenza A virus infection [34]. 12-HETE may be involved in the inflammatory reaction process after viral infection. Additionally, 15-oxoETE has been reported to inhibit the proliferation of endothelial cells [35] and to mediate the adhesion of monocytes to endothelial cells [36].

Patients with COVID-19 6 months after discharge exhibited significantly higher levels of triglycerides (TG) than healthy controls (Fig. 3B). Metabonomic studies of patients with acute COVID-19 infection have also reported the existence of abnormal levels of TG [16, 37]. We also found abnormalities in apolipoprotein levels in COVID-19 survivors 6 months after discharge; specifically, apolipoprotein D (APOD) and apolipoprotein M (APOM) levels were demonstrated to be reduced (Fig. 2B). The decrease in the levels of these two types of apolipoproteins was also found in the proteomic analysis of patients with acute COVID-19 infection [15]. Further, animal studies have found that APOD deficiency is associated with elevated levels of triglycerides [38].

### Abnormality of pulmonary fibrosis-related protein levels

Both transforming growth factor beta 1 (TGFβ1) and fibronectin 1 (FN1) are known to be drivers of pulmonary fibrosis [39]. TGFβ1 expression was downregulated, whereas FN1 expression was upregulated in COVID-19 survivors 6 months after discharge (Fig. 2B). Particularly, TGFβ1 has been found to promote the differentiation of fibroblasts into myofibroblasts that produce excessive extracellular matrix [40]. Excessive deposition of FN in the extracellular matrix is known to be a major feature of pulmonary fibrosis. The upregulation of the FN1 metabolite in COVID-19 survivors may explain the residual imaging abnormalities observed during the conduction of follow-up of patients with COVID-19 [41–43].

### Distinct profiles of severe and non-severe COVID-19 survivors

**Fig. 4:**
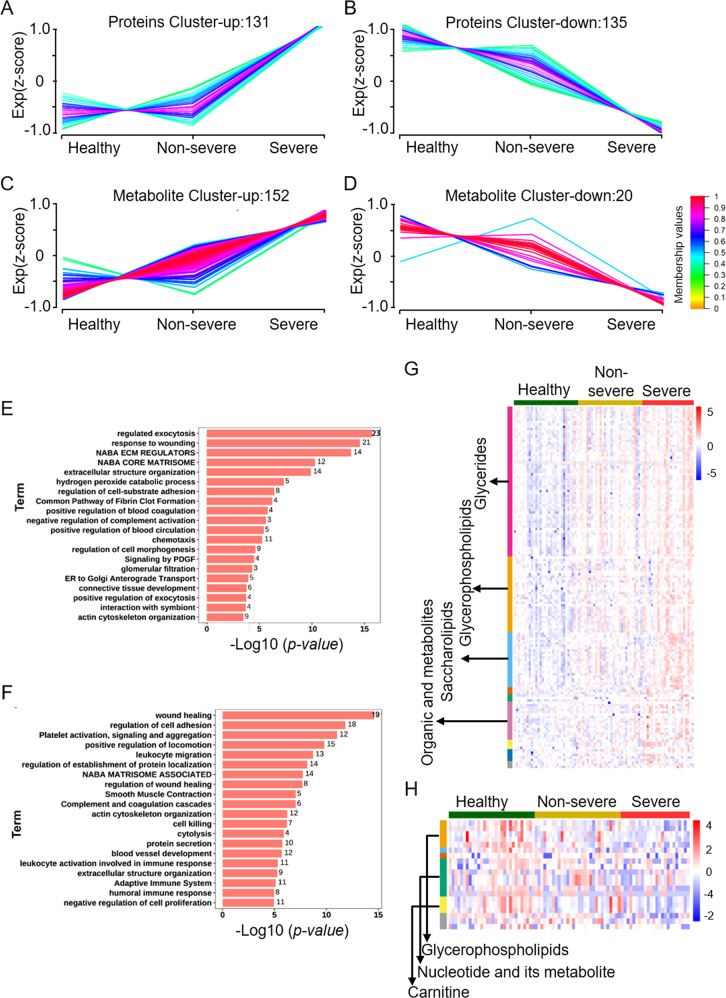
Expression profiles were analyzed according to protein and metabolic abundance between severe and non-severe COVID-19 survivors 6 months after discharge. **A**–**D** The result of cluster analysis in processing conditions (Healthy-Non-severe-Severe) by the Mfuzz package. The result of continuous up-regulation proteins (**A**) and continuous down-regulation proteins (**B**). Results of continuously up-regulated metabolites (**C**) and continuously down-regulated metabolites (**D**). Numbers of proteins and metabolites are indicated for each cluster. Color bar represents *Z* score change from −1 to 1. **E**, **F** Barplot for function enrichment result (including KEGG and GO) for up-regulated proteins (**E**, protein cluster-up) and down-regulated proteins (F, protein cluster-down) (Top20); *p* value < 0.05 was identified as significantly changed terms. The *X* axis shows the *p* value of each term, and *Y* axis shows the function terms. **G**, **H** Heatmap visualization of up-regulated metabolites (**G**, metabolites cluster-up) and down-regulated metabolites (**H**, metabolites cluster-down) under the processing conditions (healthy, non-severe, severe).

The protein and metabolic abundance matrix were processed using the Mfuzz package to reveal the presence of 6 clusters each. According to the experimental design, we only selected 2 types of clusters for analysis. We observed that a single type of clusters showed continuous and abundant upregulated (proteins: cluster-up; and metabolite: cluster-up) expression in accordance with the change in processing conditions (healthy-non-severe-severe) (containing a total of 131 proteins that were included in the P1 protein set (Fig. 4A) and 152 metabolites that were included in the M1 metabolite set (Fig. 4C)). We separately subjected proteins in the P1 protein set to functional and pathway enrichment analyses. GO and KEGG enrichment analysis results revealed that these proteins played major roles in exocytosis regulation, response to wounding, regulation of cell-substrate adhesion, positive regulation of blood coagulation, and other processes (Fig. 4E, Table S5). Hierarchical clustering results showed evident group differentiation based on multiple components, including glycerides (GL), GP, saccharolipids (SL), and organic acids and their derivatives, according to the metabolites in the set with an abundance of the M1 metabolite (Fig. 4G). We observed that another type of clusters included those whose expression levels were significantly downregulated (protein: cluster-down; and metabolite: cluster-down) (containing a total of 135 proteins that were included in the P2 protein set (Fig. 4B) and 20 metabolites that were included in the M2 metabolite set M2 (Fig. 4D)). GO and KEGG enrichment analysis revealed that these proteins played major roles in wound healing, regulation of cell adhesion and platelet activation, signaling, and aggregation (Fig. 4F, Table S6). Moreover, we found that the expression of metabolites in the M2 metabolite set, mainly including nucleotide and its metabolites (7 compounds), GP (5 compounds), carnitine (3 compounds), which were low in the severe group (Fig. 4H).

## Discussion

In this study, we analyzed the changes in plasma proteomics and metabolomics of COVID-19 survivors 6 months after discharge from the hospital and found that COVID-19 survivors exhibited significant differences in the extracellular matrix, immune response, and hemostasis pathways compared to the healthy control subjects. COVID-19 survivors presented with abnormal lipid metabolism, disordered immune responses, and changes in pulmonary fibrosis-related proteins, 6 months after discharge.

Adults often present with an overexuberant systemic inflammation in response to SARS-CoV-2 infection [44, 45], especially a severe SARS-CoV-2 infection, which is dominated by a hyperactivated/exhausted immune response [20]. Single-cell sequencing was used to determine the dynamic nature of immune responses during the progression of COVID-19 [46]. COVID-19 survivors exhibited abnormalities in immune response, complement, and hemostasis 6 months after discharge, suggesting that the immune abnormalities caused by SARS-CoV-2 were not resolved in 6 months after discharge. Arachidonic acid and other unsaturated fatty acids are proinflammatory components of the innate immune response and are known to inactivate enveloped viruses and inhibit the proliferation of various microbial organisms [47, 48]. Leukotrienes can enhance the ability of the immune system to eliminate microorganisms and to produce antibacterial agents by regulating the innate immune response [49], and may thus be potential therapeutic targets for COVID-19 [50]. The production of PGE2 was found to increase in response to COVID-19, suggesting the existence of a possible direct relationship between the levels of PGE2 and the severity of the disease [51]. Likewise, the abnormal arachidonic acid metabolites observed in our study might have an impact on the prognosis of COVID-19 survivors.

COVID-19 survivors 6 months after discharge exhibited significant lipid metabolism abnormalities. Studies have found that high levels of TG might affect the progression and prognosis of COVID-19 [52]. The level of TG in severe and non-survivors of COVID-19 was significantly higher than that in mild and survivors [53, 54]. Elevated levels of TG have been reported to reduce the level of immunoglobulin G that confers protection against COVID-19 [55]. A decrease in the plasma level of APOM was associated with impaired endothelial function, while a lack of APOM in mice caused dysfunctional endothelial barrier function in the lungs [56]. Based on these findings, lipid metabolism disorders observed in both acute and survivors of COVID-19 infections warrant extensive investigation.

Residual lung lesions, especially pulmonary fibrosis, in patients recovered from COVID-19, are noteworthy issues. Several studies have reported the occurrence of lung injury in patients with acute COVID-19 infection and early recovery [57]. The autopsy results of COVID-19 cases have also revealed the presence of alveolar damage and pulmonary fibrosis [58, 59]. Moreover, TGFβ expression was shown to be significantly increased in the serum samples of patients with acute COVID-19 infection, especially in severe type patients [60]. Furthermore, the concentration of FN did not significantly increase in the serum of patients with acute SARS-CoV-2 infection [61]. However, the mRNA and protein levels of TGFβ1 and FN1 were found to be increased in human epithelial cells after 24 h of SARS-CoV-2 infection [39]. In the present study, 26.5% of COVID-19 survivors showed lung imaging abnormalities at 6 months after discharge. While the serum proteomics showed a decrease in the levels of TGFβ1 and an increase in the levels of FN1 in COVID-19 survivors 6 months after discharge. This might be attributable to the fact that after half a year of SARS-CoV2 infection, although the profibrotic effect might have gradually weakened, the increase in FN1 levels caused by early infection might require more time to return to normalcy. Therefore, residual lesions could be observed in the follow-up imaging of patients 6 months after discharge [9]. Hence, the follow-up imaging of discharged patients with COVID-19 should be conducted for a longer period.

This study had several limitations. First, due to the limited number of follow-up patients, the sample size was small. Second, we did not perform proteomics and metabolomics correlation analysis in our patients during the phase of acute infection, and thus we could not exactly evaluate whether there were differences in proteomics or metabolomics results between these patients during the acute infection and 6 months after discharge from the hospital. However, our results show that apolipoprotein D (APOD) and APOM levels were reduced in COVID-19 survivors 6 months after discharge. A decrease in the levels of these two types of apolipoproteins was also found in severe COVID-19 patients during infection stage [15, 62]. This suggested that triglyceride metabolism is reduced in COVID-19 survivors until 6 months after discharge. Compared with the healthy individuals, the level of TGFβ1 increased in the plasma of COVID-19 patients during acute infection stage but decreased in COVID-19 survivors 6 months after discharge [62]. Nonetheless, the high level of FN1 after discharge indicated that fibrotic progression might still be a concern. In addition, leukocyte migration, extracellular matrix organization, and complement and coagulation cascades were all altered during both the acute infection stage and 6 months after discharge [14, 15].

In conclusion, findings of the present study suggest that COVID-19 survivors show persistent proteomic and metabolomic abnormalities 6 months after discharge from the hospital. Hence, the recovery period for COVID-19 survivors may be longer.

## Materials and methods

### Subjects and study design

All participants were from the Haihe Hospital (Tianjin, China). We collected a total of 84 plasma samples from 30 healthy people, 30 recovered non-severe patients and 24 recovered severe patients. All the enrolled patients met the diagnostic criteria, clinical classification, and discharge criteria of the “Chinese Clinical Guidance for COVID-19 Pneumonia Diagnosis and Treatment (7th edition)” published by the China National Health Commission. According to the clinical symptoms, confirmed patients with COVID-19 can be divided into the following 4 types: mild, moderate, severe, and critical. Mild type: mild clinical symptoms without signs of pneumonia on chest imaging; moderate type: fever and respiratory symptoms, and radiologic signs of pneumonia; severe type: any of the following four conditions: (1) shortness of breath, RR ≥ 30 times/min; (2) oxygen saturation ≤93% at rest; (3) alveolar oxygen partial pressure/fraction of inspiration O_2_ (PaO_2_/FiO_2_) ≤300 mmHg (1 mmHg = 0.133 kPa); (4) radiologic signs of significant progression of lesion ˃50% within 24–48 h; critical type: any of the following conditions: (1) respiratory failure with a necessity of mechanical ventilation; (2) shock; (3) other organ failures with a necessity of subjection to ICU monitoring and treatment. In this study, 22 severe and 2 critical patients were included and categorized as the severe group, whereas 30 moderate patients were categorized as the non-severe group. Finally, 30 age-matched medical staff from the Tianjin Haihe Hospital were recruited as healthy volunteers; subjects in this group showed negative results in nucleic acid and antibody tests for SARS-CoV-2 and without lung abnormalities (Fig. 1A).

General participant information, including age, sex, comorbidity, and clinical treatment, was collected using a standard form. Furthermore, each patient completed a symptom questionnaire regarding his or her clinical symptoms at the follow-up examination 6 months after discharge. The modified Medical Research Council (mMRC) scale and Borg scores were also collected through a combination of questionnaires, as described previously [63]. The mMRC scale was used to assess the degree of dyspnea in a variety of respiratory diseases on a scale from 0 to 4. The Borg scores showed verbal descriptions of the severity of dyspnea or fatigue, which corresponded to specific numbers for intensities [19]. High-resolution computed tomography (HRCT) of the chest was performed for 49 patients, including 28 patients in the non-severe group and 21 in the severe group using a Canon 64-slice helical CT scanner (Aquilion Prime 128, Canon Medical Systems, Otawara, Japan). The acquired chest HRCT images were interpreted by three radiologists.

### Sample collection

All enrolled subjects were strictly fasted for 12 h and were prohibited from drugs for 48 h before the collection of plasma samples. In the early morning, 5 mL of venous blood was collected and the plasma was obtained by centrifugation at 4 °C (2000 rpm, 10 min). Each plasma sample was split into four fractions, two of them for proteome analysis (library construction and sample assay) and two for metabolome analysis (hydrophilic and hydrophobic analysis). Then, all plasma samples were stored in a refrigerator at −80 °C for proteomic and metabolomic analysis (Fig. 1B).

### Sample preparation for proteome analysis

Plasma from each sample was mixed with the reaction solution buffer (1% sodium deoxycholate, 10 mM tris(2-carboxyethyl) phosphine hydrochloride, 40 mM 2-chloroacetamide). The reaction was carried out at 56 °C for 30 min for protein denaturation, disulfide bond reduction, and cysteine SH alkylation. The protein concentration was determined by Bradford method. Then, each sample was diluted with an equal volume of H_2_O, and trypsin was added at a ratio of 1:50 (enzyme: protein, w/w) and incubated overnight at 37 °C for digestion. After centrifugation (12,000 × *g*, 15 min), the supernatant was subjected to peptide purification using self-made desalting columns. The peptide eluate was vacuum-dried and stored at −20 °C until use.

### Construction of a COVID-19 plasma proteome spectral library

We constructed a COVID-19 plasma spectral library by pooling an equal amount of peptide from each sample and re-dissolving them in buffer A (2% acetonitrile, 0.1% formic acid). To ensure the cleanliness and high quality of the library, the peptide mixture was desalted again using a Gemini C18 column (5 μm, 4.6 × 250 mm) and was eluted using high-pH reverse-phase chromatography (LC-20AB liquid phase system). The gradient elution was carried out at a flow rate of 1 mL/min: 5% mobile phase B (95% acetonitrile, pH 9.8) for 10 min, 5–35% for 40 min, 35–95% for 1 min, 100% for 3 min, 5% mobile phase B equilibrated for 10 min. The elution peak was monitored at 214 nm and a fraction was collected every minute. Eluates were concatenated into 54 fractions, with which two or three fractions every 20 fractions were mixed. For the resulting 20 fractions, each fraction was then analyzed in the data-dependent acquisition (DDA) mode to construct a COVID-19 plasma spectral library.

### Liquid chromatography-mass spectrometry for proteome analysis

The peptides were redissolved in mobile phase A (2% acetonitrile, 0.1% formic acid), centrifuged at 20,000 × *g* for 10 min and the supernatant was separated using an UltiMate 3000 UHPLC system (Thermo, USA). Briefly, the peptides entered the trap column for enrichment, then entered the connected self-packed C18 column (1.8 μm, 150 μm × 350 cm) and were separated at a flow rate of 500 nL/min. The peptides were eluted using the following gradient: 0–5 min, 5% mobile phase B (98% acetonitrile, 0.1% formic acid); 5–90 min, 5–25% mobile phase B; 90–100 min, 25–35% mobile phase B; 100–108 min, 35–80% mobile phase B; 108–113 min, 80% mobile phase B; 113–120 min, 5% mobile phase B. The peptides separated by liquid phase were ionized using a nano ESI source and then connected to a Q-Exactive HF tandem mass spectrometer (MS) (Thermo, USA).

To construct a COVID-19 plasma proteome spectral library, the Q-Exactive HF instrument was operated in the DDA mode. The m/z range of MS1 was 350–1500, the resolution was 120,000, and the maximum ion injection time (MIT) was 100 ms. The top 20 precursors were selected for the MS/MS experiment by higher-energy collision dissociation (HCD) with a resolution of 30,000, MIT of 100 ms, and dynamic exclusion time of 30 s. The automatic gain control (AGC) was MS 3e6, MS/MS 1e5.

To analyze the plasma proteome in each subject, the Q Exactive HF instrument was operated in the data-independent acquisition (DIA) mode to switch between full-scan MS and MS/MS acquisition. The m/z range of MS1 was 400–1250, with a resolution of 120,000 and an MIT of 50 ms. All precursor ions were selected for collision cells for fragmentation by HCD. The MS/MS resolution was set at 30,000, the maximum fill time at automatic, and the AGC target at 1e6. DIA was performed with a variable isolation window, with 45 windows in total.

For quality control (QC) of the proteomic analysis, 10 μL of each sample was pooled as a QC sample. Then, 10 QC samples were randomly evaluated by calculating the CV of proteins in the QC samples.

### Protein identification and quantitation

DDA data were identified using MaxQuant (version 1.5.3.30) [64]. The reference database sequences were obtained from the UniProt *Homo sapiens* proteome database (172,419 sequences). The spectral library was built using peptide/protein entries that satisfied a false discovery rate (FDR) ≤ 1%. Carbamidomethyl (C) was set as a fixed modification, and oxidation (M) and acetyl (protein N-term) were set as variable modifications. DIA data were processed using Spectronaut software (Biognosys, https://biognosys.com/shop/spectronaut) [65] against the self-built plasma spectral library to achieve deeper proteome identification and quantification. The FDR was estimated using the mProphet scoring algorithm with 1% FDR control at the peptide-spectrum match, peptide, and protein levels. Next, the R package Msstats was used for log2 transformation, normalization, and *p* value calculation of the data [66]. Differentially expressed proteins (DEPs; *p* < 0.05 and fold-change ≥1.5 or *p* < 0.05 and fold-change <0.67) were identified for further analysis. The *p* values were then adjusted using the Benjamini-Hochberg correction (*p* adjust <0.05). The DEPs are listed in Supplementary Table S1.

### Extraction of hydrophilic and hydrophobic compounds for metabolome analysis

To detect the maximum possible metabolites, both the hydrophilic and hydrophobic metabolites were respectively extracted and analyzed as per a previously reported method [67]. To extract hydrophilic compounds, the plasma samples were thawed on ice and vortexed for 10 s. Six volumes of pure methanol was added to one volume of plasma samples, the mixture was thoroughly mixed for 3 min and centrifuged (10 min at 12,000 rpm, 4 °C). The supernatant was collected and centrifuged again (5 min at 12,000 rpm, 4 °C). The final supernatant was collected for LC-MS/MS analysis.

To extract hydrophobic compounds, the plasma samples were thawed on ice, vortexed for 10 s, and centrifuged (5 min at 3000 rpm, 4 °C). Then, the plasma samples were thoroughly mixed with 1 mL of lipid extract mixture (methanol, *tert*-butyl methyl ether, and internal standard mixture) for 15 min. This mixture was added with 200 μL of water, vortexed for 1 min, and centrifuged again (10 min at 12,000 rpm, 4 °C). The supernatant was extracted and concentrated, dissolved in 200 μL mobile phase B (acetonitrile/isopropanol (10%/90%, v/v) containing 0.04% acetic acid and 5 mM ammonium formate) and subjected to LC-MS/MS analysis.

### Ultra-performance liquid chromatography-tandem mass spectrometry (UPLC-MS/MS)

The hydrophilic compounds were injected into a Waters ACQUITY UPLC HSS T3 column (1.8 µm, 2.1 mm × 100 mm). The column temperature, flow rate, and injection volume were 40 °C, 0.4 mL/min, and 2 μL, respectively. The mobile phase consisted of water containing 0.1% formic acid (A) and acetonitrile containing 0.1% formic acid (B). The gradient was as follows: from 5% B to 90% B in 11 min, then held for 1 min, and finally decreased to 5% B for 2 min. Mass spectrometric scans were acquired with a 6500+ QTRAP^®^ LC-MS/MS System equipped with an electrospray ionization (ESI) Turbo Ion-Spray interface, operating in positive and negative ion mode and controlled by Analyst 1.6.3 software (Sciex). The ESI source operation parameters were as follows: source temperature was 500 °C; ion spray voltage was 5500 V in positive ion mode (or −4500 V in negative ion mode); ion source gas I, gas II, and curtain gas set at 55, 60, and 25 psi, respectively; and collision-activated dissociation (CAD) set to high.

Meanwhile, the hydrophobic compounds were injected into a Waters Accucore^TM^ C30 column (2.6 µm, 2.1 mm × 100 mm). The column temperature, flow rate, and injection volume were 45 °C, 0.35 mL/min, and 2 μL, respectively. The mobile phase consisted of acetonitrile/water (60%/40%, v/v, 0.1% formic acid, 10 mmol/L ammonium formate) (A) and acetonitrile/isopropanol (10%/90% v/v, 0.1% formic acid, 10 mmol/L ammonium formate) (B). The gradient was as follows: from 20 to 95% B in 15.5 min, then held for 2 min, and finally decreased to 20% B for 2.5 min. Mass spectrometric scans were acquired with a 6500+ QTRAP^®^ LC-MS/MS System equipped with an ESI Turbo Ion-Spray interface, operating in positive and negative ion mode and controlled by Analyst 1.6.3 software (Sciex). The ESI source operation parameters were as follows: source temperature was 500 °C; ion spray voltage was 5500 V in positive ion mode (or −4500 V in negative ion mode); ion source gas I, gas II, and curtain gas set at 45, 55, and 35 psi, respectively; and CAD set to medium.

Instrument tuning and mass calibration were performed with 10 and 100 μmol/L polypropylene glycol solutions in the triple quadrupole (QQQ) mode. Based on the self-built database and metabolite information in the public database, the materials were qualitatively analyzed according to the secondary spectrum information and the isotope signal was removed during the analysis. QQQ scans were acquired as multiple reaction monitoring (MRM) experiments with the collision gas (nitrogen) set to 5 psi [68]. The de-clustering potential (DP) and collision energy (CE) for individual MRM transitions were obtained with further DP and CE optimization. The quantification of metabolites was accomplished using the targeted MRM approach [69]. A specific set of MRM transitions were monitored for each period according to the metabolites within this period. Each sample analysis was conducted on both the positive and the negative modes, and the MRM transitions are listed in Supplementary Table S2.

### Metabolite identification and quantitation

The MS data were processed using Software Analyst 1.6.3. The repeatability of metabolite extraction and detection was evaluated using the total ion current (TIC) and multiple peaks of MRM. Qualitative analysis of the first-order and second-order spectra detected by mass spectrometry was carried out on the basis of a home-made metadata database and existing metabolomic databases, including MassBank (http://www.massbank.jp/) [70], HMDB (http://www.hmdb.ca/) [71], LIPID MAPs (www.lipidmaps.org/data/structure/) [72, 73] and Metlin (http://metlin.scripps.edu/index.php) [74].

For the quality control (QC) of metabolomic analysis, we pipetted 10 μL of each sample to pool a QC sample. One QC sample was analyzed after every 10 samples in the LC-MS/MS running sequence. The CV value of the peak area for each metabolite in QC samples was calculated to evaluate the stability of the LC-MS/MS analysis. The quantitation of metabolites was accomplished using MRM triple quadrupole mass spectrometry. The intensity-based abundances of each metabolite were calculated by e peak area of each chromatographic peak. After obtaining the mass spectral analysis data of different samples, the peak areas of all the mass spectral peaks were integrated, and the mass spectral peaks of the same metabolites in different samples were integrated and corrected. We calculated the CV values of the metabolites in the QC sample, and metabolites whose CV values were larger than 0.5 were excluded.

The supervised multivariate method, PLS-DA, was used to resolve the metabolome differences among the two groups (Brereton RG et al., 2014). The relative importance of each metabolite to the PLS-DA model was checked using the parameter called variable importance in projection (VIP). Based on the VIP of the PLS-DA model, the *p* value or fold change of univariate analysis was applied to further screen the differential metabolites [75]. The screening criteria were as follows: fold change ≥2 or fold change ≤0.5, *p* < 0.05, and VIP ≥ 1. If the above three conditions were satisfied, the metabolite was considered significantly different between the groups. DEMs are listed in Supplementary Table S3.

### Pathway analysis

Using the Gene Ontology (GO, http://geneontology.org/) and the Kyoto Encyclopedia of Genes and Genomes (KEGG, http://www.genome.jp/kegg/) databases, we performed GO and KEGG pathway enrichment analysis based on differential proteins and metabolites to explore the biological process of the disease [76, 77]. The GO analysis included biological process (BP), cellular component (CC), and molecular function (MF) as the three main categories. A *p* < 0.05 was considered significant. Cluster analysis of protein expression or metabolite intensity from the different patients was performed using Mfuzz v.2.46.0 [78], which can identify underlying time-series patterns of expression profiles and cluster proteins or metabolites with similar patterns to clarify the dynamic patterns of proteins or metabolites and their functional linkages.

### Statistical analysis

Principal component analysis (PCA) and hierarchical cluster analysis were performed using the distance matrix calculated using the R statistical language (v3.6.1; https://CRAN.R-project.org) [79], pheatmap (Version 1.0.12, https://cran.r-project.org/web/packages/pheatmap/index.html), and ggord (Version 1.1.5). The normality of the data distributions was assessed using the Kolmogorov-Smirnov test. Normally distributed data are presented as the mean (± standard deviation), whereas abnormally distributed data are presented as the median (± interquartile range), and categorical variables are presented as frequencies (%). Differences between groups were analyzed using Student’s *t* test or Mann–Whitney test (for continuous data) and Fisher’s exact test or chi-squared test (for categorical data). The statistical significance was calculated for reserved proteins and metabolites using the unpaired two-sided Student’s *t* test, and the adjusted *p* value was calculated using the Benjamini-Hochberg correction (adjusted *p* < 0.05).

## Supplementary information

Legends to supplementary figures

Figure S1

Figure S2

Figure S3

Figure S4

Supplementary tables

Checklist

## Data Availability

The data that support the findings of this study will be available from the corresponding author upon reasonable request. The MS proteomics and metabolomics data have been deposited to the ProteomeXchange Consortium (http://proteomecentral.proteomexchange.org) via the iProx partner repository [80] with the dataset identifier PXD025148 (https://www.iprox.org//page/project.html?id=IPX0002924000).
