# Supplementary material for: Plasma proteomic and metabolomic characterization of COVID-19 survivors 6 months after discharge
Source: Cell Death Dis. 2022 Mar 14;13(3):235. doi: 10.1038/s41419-022-04674-3 (PMC8919172; doi:10.1038/s41419-022-04674-3)
Supplement: Supplementary file 1 — Legends to supplementary figures [file 41419_2022_4674_MOESM1_ESM.docx]

**Legends to supplementary figures**

**Fig S1. Quality control (QC) of proteomic and metabolomic data.**

A. Distribution of coefficient variation (CV) values in the QC samples for protein. The CV values of 92% proteins were less 30%.

B. Distribution of CV values in the QC samples for metabolite. The CV values of 82% and 95% metabolites were less than 20% and 30%, respectively.

C. CV of the proteomic data calculated using the proteins quantified in four QC samples with pooled samples from all samples. CV of the metabolomic data was calculated from nine QC samples.

**Fig S2.** **Differential protein analysis of plasma samples obtained from COVID-19 survivors 6 months after discharge and healthy control subjects.**

A. Volcano plots showing the change in the transformed adjusted *p-value* (-log10) against the log2(fold change). Blue dashed lines: cut-off values (Log2|fold change| >1.5 and adjusted *p-value* <0.05). Red dots: highly expressed protein in COVID-19 survivors. Purple dots: low expression of protein molecules. Important proteins are labeled. The size of the points varies according to the absolute value of (Log2(fold change)).

B. PCA plot of differentially expressed proteins (DEPs). Green, yellow, and red data points are healthy control subjects and non-severe and severe COVID-19 survivors at 6 months after discharge, respectively.

**Fig S3.** **Enrichment analysis of differentially expressed proteins (DEPs) from COVID-19 survivors at 6 months after discharge and healthy control subjects.**

A. Network of enriched function terms. Different colors represent different function terms. The enrichment analysis focused on three pathways: immunological response (blue dashed line), hemostasis (green dashed line), and ECM (red dashed line).

B. Bar plot of functional enrichment results (including KEGG and GO) for DEPs; a *p-value* < 0.05 was considered significant. The X-axis shows the enrichment score (-log10(hypergeometric test *p-value*)) of each term, and the Y-axis shows the function terms. Different colors represent different function terms. Immunological response (blue), hemostasis (green), and ECM (red).

**Fig S4.** **Differential altered metabolites analysis of plasma samples obtained from COVID-19 survivors 6 months after discharge and healthy control subjects.**

A. Volcano plots showing the change in the transformed adjusted *p-value* (-log10) against the log2(fold change). Blue dashed lines: cut-off values (Log2|fold change| >2 and adjusted *p-value*<0.05). Red dots: highly expressed metabolites in COVID-19 survivors. Purple dots: low expression of metabolites in COVID-19 survivors. Important metabolites present are labeled. The size of the points varies according to the absolute value of (Log2(fold change).

B. PCA plot of differentially abundance metabolites (DEMs). Green, yellow, and red data points are healthy control subjects, non-severe, and severe COVID-19 survivors 6 months after discharge, respectively.

C. Classification of DEMs identified in this study. Different block represents different class metabolites. The blue block represents glyceride (103 compounds), which were the most upregulated metabolites in COVID-19 survivors at 6 months after discharge. The red block represents oxidized lipids, which were mainly down-regulated in COVID-19 survivors at 6 months after discharge.

D. Bar plot of important differentially altered metabolites. The X-axis shows the ID of different metabolites and the Y-axis shows the VIP value.
