# Supplementary figures and images for: Plasma proteomic and metabolomic characterization of COVID-19 survivors 6 months after discharge
Source: Cell Death Dis. 2022 Mar 14;13(3):235. doi: 10.1038/s41419-022-04674-3 (PMC8919172; doi:10.1038/s41419-022-04674-3)

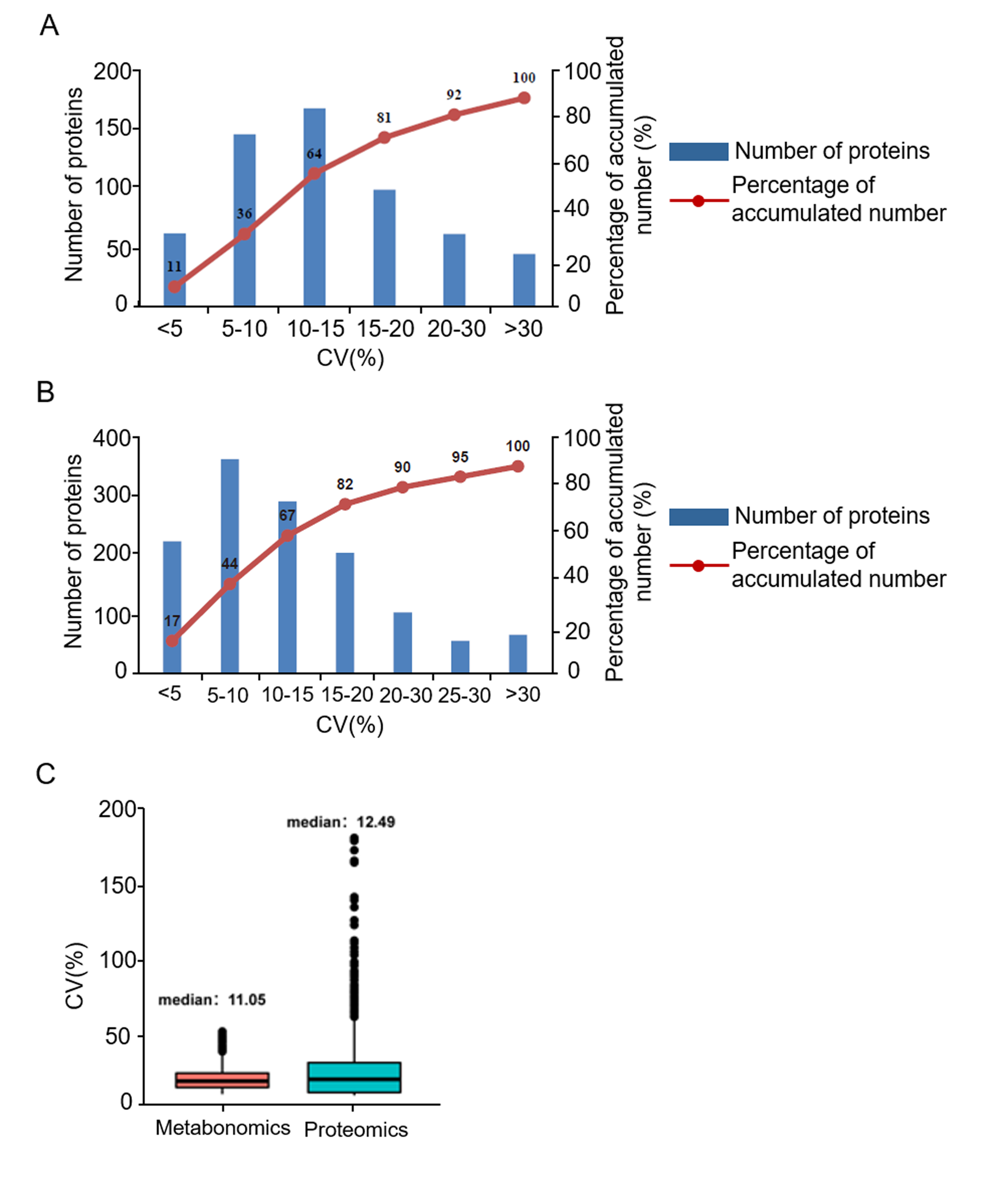

Supplement: Supplementary file 2 — Figure S1 [file 41419_2022_4674_MOESM2_ESM.tif]

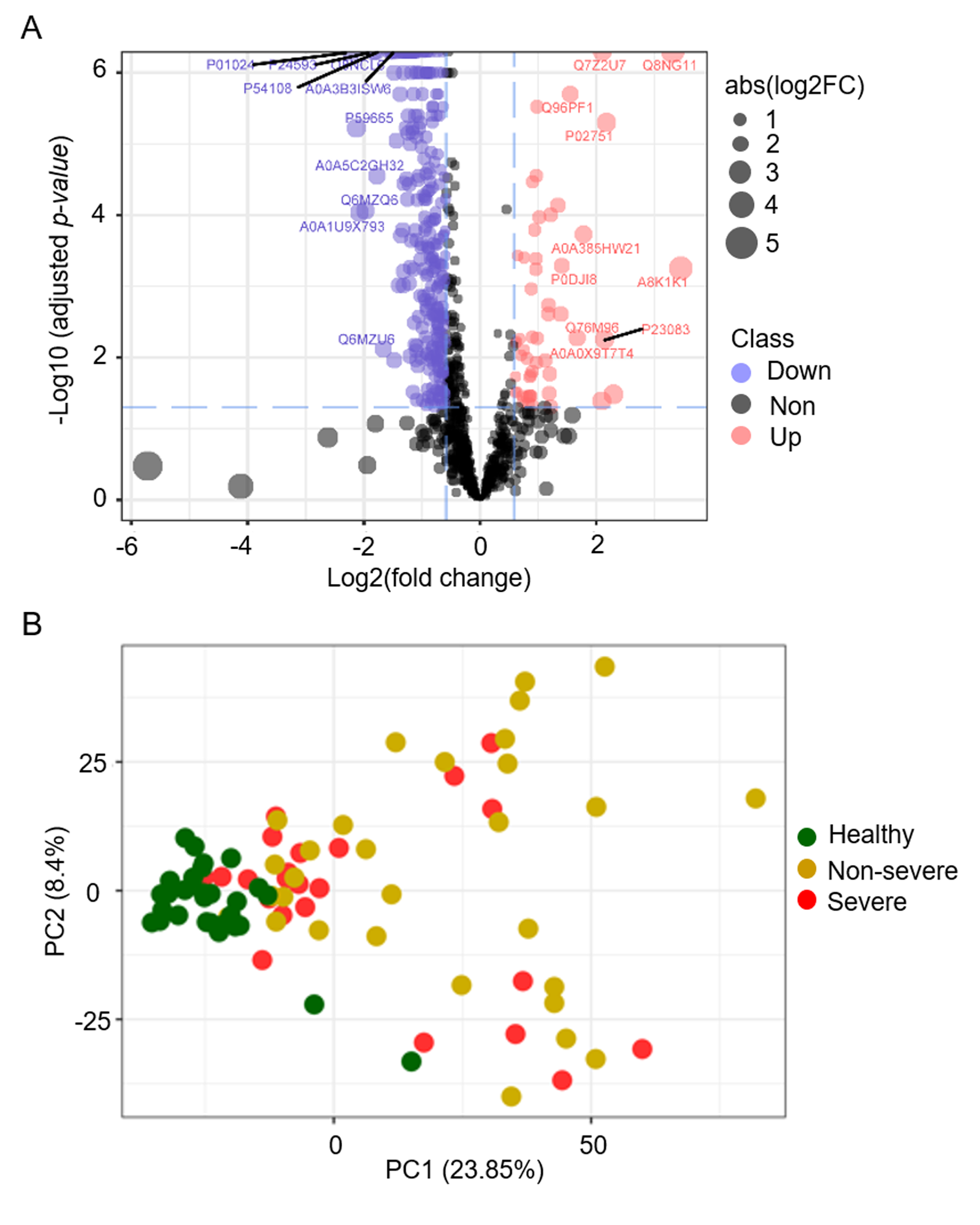

Supplement: Supplementary file 3 — Figure S2 [file 41419_2022_4674_MOESM3_ESM.tif]

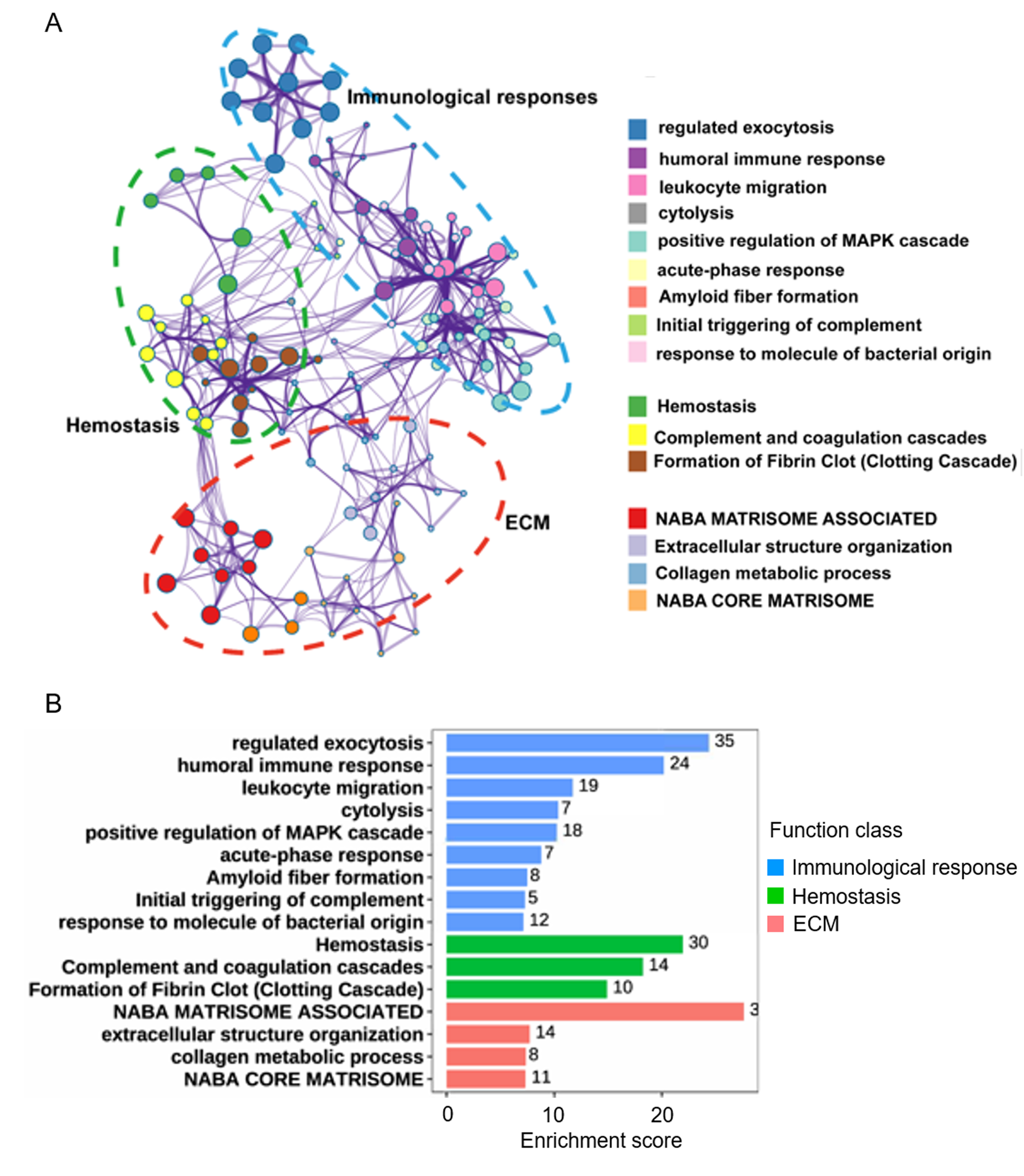

Supplement: Supplementary file 4 — Figure S3 [file 41419_2022_4674_MOESM4_ESM.tif]

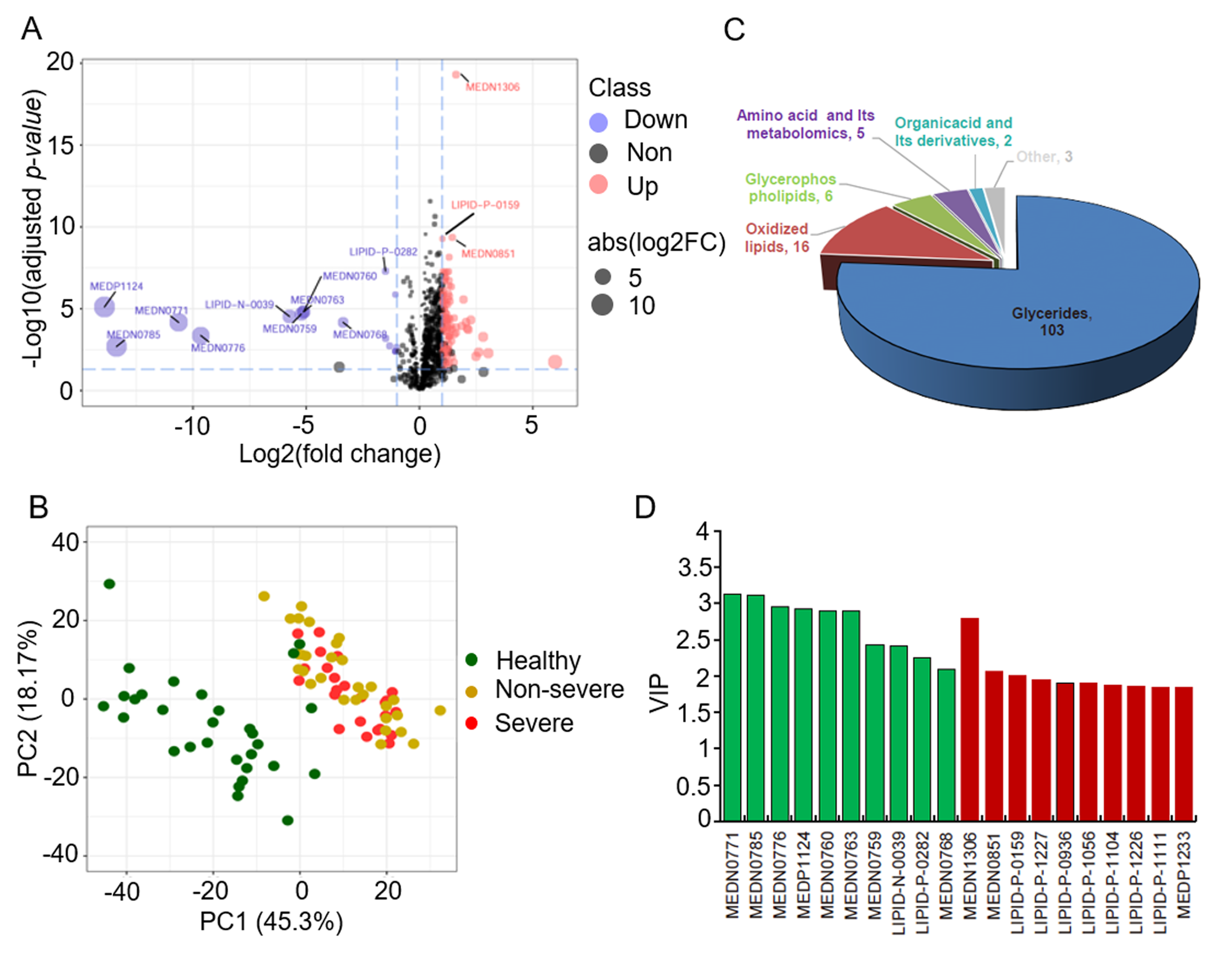

Supplement: Supplementary file 5 — Figure S4 [file 41419_2022_4674_MOESM5_ESM.tif]
